# Developing a Quality Improvement Framework to Enhance the Health System User Experience for Individuals Living With Type 1 Diabetes: The Reshape T1D Study

**DOI:** 10.1111/hex.70172

**Published:** 2025-02-05

**Authors:** Jamie Boisvenue, Youssef A. Elezzabi, Kim Young, Kathleen Gibson, Heather Hinz, Reid McClure, Robyn Homulos, Jude Spiers, Peter Senior, Roseanne Yeung

**Affiliations:** ^1^ Department of Medicine Faculty of Medicine & Dentistry, College of Health Sciences, University of Alberta Edmonton Alberta Canada; ^2^ Alberta Diabetes Institute, University of Alberta Edmonton Alberta Canada; ^3^ Alberta Physician Learning Program, University of Alberta Edmonton Alberta Canada; ^4^ Patient Co‐Researcher University of Alberta Edmonton Alberta Canada; ^5^ Faculty of Pharmacy and Pharmaceutical Sciences, College of Health Sciences, University of Alberta Edmonton Alberta Canada; ^6^ Alberta Health Services Edmonton Calgary Alberta Canada; ^7^ Patient Co‐Researcher University of Alberta Edmonton Calgary Alberta Canada; ^8^ Alberta Health Services Edmonton Alberta Canada; ^9^ Faculty of Nursing, College of Health Sciences, University of Alberta Edmonton Alberta Canada

**Keywords:** community‐based participatory research, diabetes, endocrinology, patient‐centered care, qualitative, type 1 diabetes

## Abstract

**Introduction:**

User experience design aims to create products and services that are accessible, usable, and enjoyable. The Reshape T1D study aims to apply these principles to understand how individuals living with T1D interact with and experience healthcare to inform T1D clinical quality improvement.

**Methods:**

Using a community‐based participatory research design, we involved four patients and four clinicians as co‐researchers throughout the research. A questionnaire and virtual semi‐structured interview were applied across a purposeful sample of 41 adults living with T1D across Alberta, Canada, between September 2021 and May 2022. Audio recordings were transcribed verbatim and de‐identified before coding. Thematic analysis was conducted on coded participant discourse through multiple coders.

**Results:**

Participants indicated the need for a centralized hub that provides consistent, reliable, and up‐to‐date T1D education and resources and an emphasis on access to mental health resources within T1D care settings. Providing greater flexibility for appointment types (ie. in‐person, virtual, etc.) and after‐hours access contributed to better self‐management and prevented emergency room visits. Participants desired a choice as to who comprises their T1D care team and for teams to address patient needs specific to their reality. We identified that medical trauma had long‐term impacts on perceptions of healthcare and contributed to a reluctance to seek future care. Women expressed challenges in discussing reproductive health with their clinicians. Diabetes online communities provide an adjunct to clinical care through peer support. Cost and access to the latest technology are ongoing barriers for many participants, especially concerning publicly funded programmes that use advanced insulin pump therapy, continuous glucose monitoring, and automated insulin delivery systems. A quality improvement framework emerged through data analysis, and findings were synthesized into actionable recommendations for ongoing clinical quality improvement.

**Conclusion:**

Our findings highlight how important health system user suggestions are for more equitable, accessible, and empathetic healthcare for individuals living with T1D. Further work is needed to explore health system user experiences with clinicians and healthcare administrators to effectively carry out T1D clinical quality improvement.

## 1 Introduction

Type 1 diabetes (T1D) is an autoimmune disease that requires insulin to regulate blood glucose levels and sustain life [1]. Individuals living with T1D must diligently monitor blood glucose and adjust insulin dosages to reduce the risk of diabetes complications. Insulin dosing is complex, requiring calculations that consider many factors [2], and both hyperglycaemia and hypoglycemia from T1D can lead to serious complications [1, 3]. Despite advances in diabetes technology and disease‐modifying therapies, many continue to struggle to meet clinical targets due to the complexities of daily management, healthcare disparities, and limited access to providers [4]. Patient‐centred care improves health outcomes and satisfaction [5], but existing quality improvement (QI) frameworks are often too general and lack specific guidance on operationalizing patient‐centred approaches [6].

Patient‐centred approaches are established in paediatric T1D care but are less explored in adults [7, 8]. Once considered a childhood condition, recent data show similar rates in adults and children [9]. As complications increase with age, there is a need to better understand adult T1D care experiences to inform quality improvement [10, 11]. Quality improvement in health services design relies on patient‐researcher partnerships that view lived experience as expertise [12, 13]. This is achieved through a shift in the research paradigm by involving patients as proactive partners in the research process rather than passive participants [14, 15]. Prioritizing issues relevant to lived experience aids in the co‐design of impactful research [16, 17, 18]. The Reshape T1D study aims to address the challenges facing adults living with T1D in navigating health services by identifying systemic barriers and co‐developing a quality improvement framework and a list of actionable recommendations to inform patient‐centred care.

## 2 Objective

The aim of this study was to (1) understand the adult T1D health system user experience in the province of Alberta, Canada and (2) to co‐develop a quality improvement framework and provide actionable recommendations for clinical quality improvement.

## 3 Materials & Methods

### 3.1 Research Design

Our study was guided by a constructivist and relativistic lens. Given that the primary researcher does not live with T1D, individuals with T1D lived experience and clinicians working in T1D care were engaged as co‐researchers to guide the entire research process. Therefore, we used a community‐based participatory research (CBPR) design by involving four patients and four clinicians as co‐researchers to co‐design, co‐lead, and co‐author the entire research process [19]. Two tools for data collection were co‐designed (a questionnaire (Table 1) and a semi‐structured interview protocol (Table 2)) over a series of twelve iterative sessions between September 2020 and August 2021. The questionnaire was administered electronically via the Research Electronic Data Capture Software (RedCAP). The semi‐structured interview was conducted one‐on‐one via Zoom.

**Table 1:** Participant Questionnaire.

General Demographic Questions	
First/Last Name	
Age: (Years)	
Biological Sex:	Female, Male, PNTS, Other (Specify)
Gender:	Cisgender, Non‐binary, Transgender, PNTS, Other (Specify)
Sexual Orientation:	Heterosexual
	Homosexual
	Bisexual
	PNTS
	Other (specify)
Ethnicity:	African
	Caucasian
	Indigenous
	East Asian/Pacific Islander
	South Asian
	Hispanic/Latino
	Middle Eastern
	PNTS
	Other (specify)
First Language	
Degree of Rurality:	Urban or Rural
Education:	Did not complete high school
	High School Diploma or equivalent
	Trade/Technical/certificate training
	Undergraduate
	Graduate or Professional Degree
	Other
Employment:	Full time
	Part time
	Temporary/Contract
	Maternity/Paternity Leave
	Unemployed
	Retired
Insurance status:	Uninsured, or insured by the employer or third party

Diabetes Specific Questions	
Age at Diagnosis	
Time of last A1c:	within 6 months, 1 year, more than 1 year, or I do not know
Latest A1c measure	
Complications:	Eye damage
	Kidney damage
	Nerve damage
	Gastroparesis
	Sexual dysfunction
	Heart disease
	Stroke
	I have no complications
	Other (specify)
Mode of Insulin	Multiple daily injections
	insulin pump
	I do not take insulin
	Other (specify)
Alberta IPTP	Yes, No, or I do not know
Blood glucose	Strips
	Continuous glucose monitor
	Flash glucose monitor
	I do not monitor my blood sugar
Healthcare Provider	Psychologist
	Pharmacist
	Dietitian
	Nurse
	Endocrinologist/diabetes specialist
	Family doctor
	Other
Last time seeking care	Within the last 3 months
	Within the last 6 months
	Within the last year
	Within the last 3 years
	More than 3 years
Frequency of care	Once every 3 months, 6 months, once a year, once every 2 years, once every 3 years

*PNTS, Prefer not to say; IPTP, Insulin Pump Therapy Program*.*

**Table 2:** Co‐developed semi‐structured interview guide.

Category 1: Appointments
1. Describe a diabetes healthcare appointment that is stuck in your memory. 2. What would your version of the best diabetes healthcare look like to you? 3. Have you ever skipped or delayed a diabetes appointment, and why? 4. Describe what you would change about your diabetes appointments that would better fit your needs and lifestyle?
Category 2: Barriers
1. What does your diabetes team do to earn your trust? 2. How does the financial cost of managing your diabetes impact your life?
Category 3: Adaptability & Resilience
1. What advice would you have for someone who is newly diagnosed? 2. What do you need from people in your life to understand the most about your diabetes? 3. Describe what it is like to talk about sensitive topics with your diabetes team.
Category 4: Influential Healthcare Providers
1. Can you think of a healthcare provider that stood out for you in terms of providing the best care, and what was it that made them stand out? 2. Can you think of a healthcare provider that stood out for you in terms of providing the poorest care, and what made them stand out?
Category 5: Support Systems
1. Describe a moment where you felt totally supported within a healthcare appointment. 2. Describe where you feel the healthcare system is failing you. 3. Do you know anyone else who is living with T1D, and what was/is that like for you?
Category 6: Therapies & Accessibility
1. What would make it easier for people living with diabetes to learn more about how to manage diabetes? 2. How would you improve the quality of diabetes education? 3. Where do you find that your diabetes team could use more training? 4. What information do you expect to receive from your diabetes team?
Category 7: Sex & Gender
1. How has your sex impacted the care that you receive from your diabetes team? 2. How do you think your diabetes team could improve care to suit your needs based on your sex? 3. How has your gender impacted the care that you receive from your diabetes team? 4. How do you think your diabetes team could improve care to suit your needs based on your gender?

To ensure that the insights we gather are informed by those living with T1D, we purposefully recruited individuals through a combination of methods, including the Diabetes Action Canada ConnecT1D recruitment portal (www.ConnecT1D.ca) [20], local TV news, social media (Twitter and Facebook), the study website (www.ReshapeT1D.com) [21], and snowball sampling. Study participants were included if they were residents of Alberta, Canada, at least 18 years of age, with a duration of T1D for at least 3 years, and had access to Zoom/telephone as this study was conducted at the beginning of the COVID‐19 pandemic restrictions. Audio and video recordings were collected, cleaned, de‐identified, and transcribed verbatim using Otter. ai. Line utterances from interviewees were parsed in RStudio (2023.09.1 + 494) to assign utterance identifiers (UIDs).

### 3.2 Coding & Analysis

**Figure 1:**
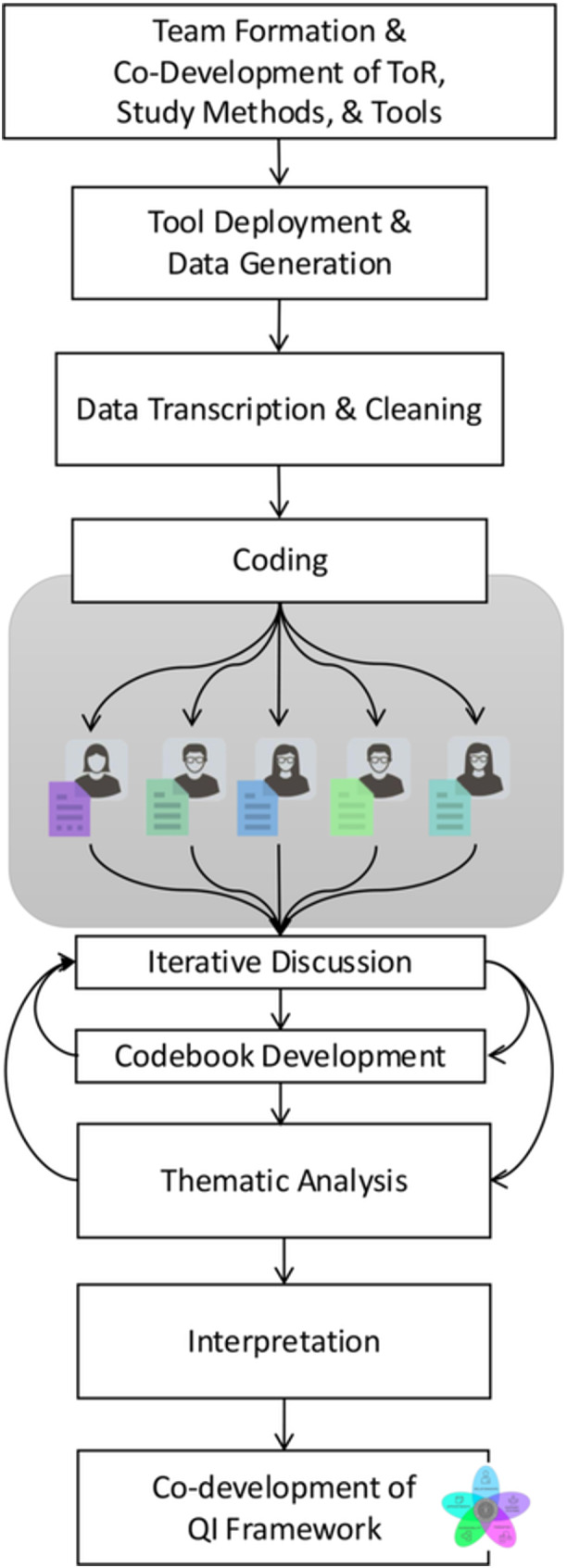
Research and Coding Process. Flow diagram of the research process, including patient and clinician engagement, code triangulation, and co‐development of the framework. *Terms of Reference (ToR), Quality Improvement (QI).

Data from the questionnaire was analyzed using descriptive statistics using means (+/‐ standard deviation, SD) for normally distributed data and median (interquartile range, IQR) for non‐normally distributed data. The semi‐structured interviews were analysed via thematic analysis with five coders (two patients, two clinicians, and the primary researcher) who were given a sample of five transcripts and asked to code inductively and independently. Coding was conducted using the Reproducible Open Coding Kit (ROCK) [22]. A tentative code structure was drafted through a series of iterative discussions. Codebook consensus was identified through code consistency across coders. Full coding then commenced, including additional codes where applicable. Themes were collated through iterative discussions with co‐researchers for final interpretation [23]. Figure 1 illustrates the research and coding process. Thematic analysis is enhanced by CBPR through the iterative co‐identified themes that can be directly translated into actionable recommendations, as shown elsewhere [24]. A detailed account of the themes is presented below, illustrated with participant quotations, identification number, sex, age, and the unique utterance identifier. Clarifications or de‐identifications are represented by “[]”.

## 4 Results

### 4.1 Participant Characteristics

Forty‐one adults living with T1D across Alberta participated in interviews between September 2021 and May 2022. The mean age of participants was 43.8 ± 15.6 years, the median age at diagnosis was 16.4 years (IQR 2.9, 29.9), and the mean duration of diabetes was 27.3 ± 17.1 years. The mean haemoglobin A1c was 7.1% ± 0.9. Most participants were female (65.9%), speaking English as a first language (92.7%), Caucasian (87.8%), residing in an urban area (82.9%), and with a post‐secondary degree (73.1%). We found that 39.0% were employed full‐time and had health benefits through their employer. Insulin administration modalities varied, with 58.5% using an insulin pump, 36.6% using multiple daily injections, and 4.9% using both. Among those using a pump, over half (56.1%) were registered in the Alberta Insulin Pump Therapy Program (IPTP), a government‐subsidized programme that provides coverage for insulin pumps and supplies for eligible patients. Most participants reported using a continuous glucose monitor (CGM) (82.8%) (Table 3).

**Table 3:** Baseline Characteristics.

Characteristic	N = 41	(%)
Age (mean years, SD)	43.8	(15.6)
Sex		
Female	27	(65.9)
Male	14	(31.4)
Age at Diagnosis (median, IQR)	16.4	(2.9, 29.9)
Duration of Diabetes (mean, SD)	27.3	(17.1)
A1c (mean %, SD)	7.1	(0.9)
English	38	(92.7)
Caucasian	36	(87.8)
Urban	34	(82.9)
Educated to Degree Level	30	(73.1)
Employment		
Full Time	16	(39.0)
Part Time	9	(22.0)
Retired	8	(19.5)
Unemployed	7	(17.0)
Temporary/Contract	1	(2.4)
Insurance		
Employer	16	(39.0)
Third Party	12	(29.3)
Partner	5	(12.2)
Uninsured	4	(9.8)
Employer & Third Party	3	(7.3)
Employer & Partner	1	(2.4)
Insulin Modality		
Insulin Pump	24	(58.5)
Multiple Daily Injections	15	(36.6)
Both	2	(4.9)
Alberta Insulin Pump Program		
Yes	23	(56.1)
No	18	(43.9)
Glucose		
CGM	17	(41.4)
Strips & CGM	17	(41.4)
Strips	7	(17.1)

### 4.2 The Type 1 Diabetes Lived Experiences Framework for Clinical Quality Improvement

**Figure 2:**
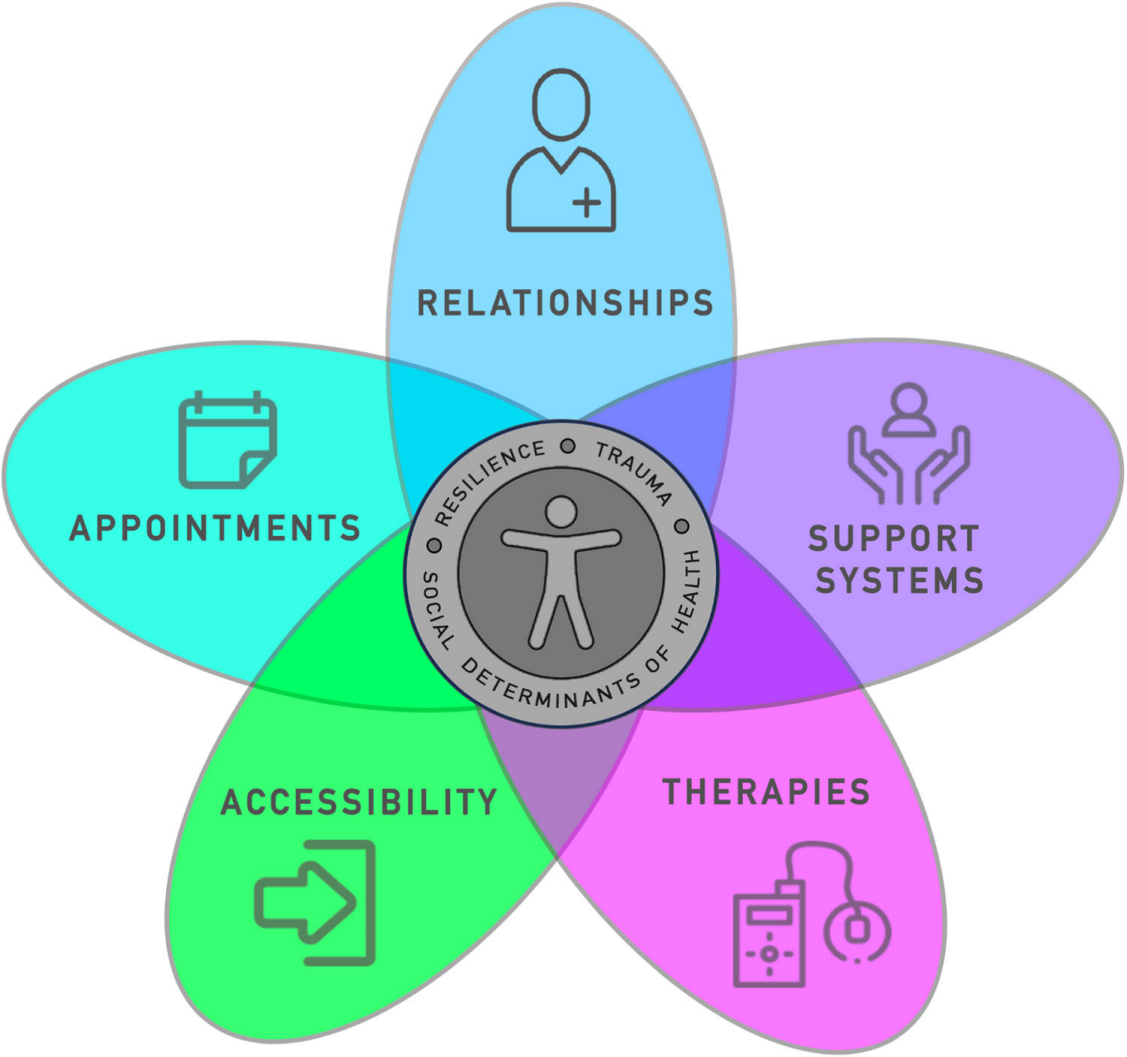
Type 1 Diabetes Lived Experiences Framework for Clinical Quality Improvement. The framework illustrates key aspects of person‐centred care for type 1 diabetes as voiced by people living with type 1 diabetes. The central circle represents the individual living with type 1 diabetes, and the surrounding ring depicts elements of lived experience that should be considered when caring for the person. Resilience refers to the strength acquired through managing and living with type 1 diabetes daily. Trauma signifies the challenges encountered in life and in healthcare interactions. Social determinants of health are the non‐medical factors that influence health and well‐being including income, education, ethnicity, and built environment. The surrounding domains that intersect with the centre represent the environmental factors that influence an individual's healthcare experience related to type 1 diabetes.

The T1D Lived Experiences Framework for Clinical Quality Improvement (QI) is an empirically derived framework co‐developed through thematic analysis of participant narratives. (Figure 2). This framework is intended to help clinicians, administrators, and policymakers understand patient‐voiced needs and desires to meaningfully inform T1D clinical quality improvement. The lived experience “ring” represents the aspects of a person that clinicians and healthcare administrators need to consider when providing health services, which include the following: experiences of resilience, history of medical or other life trauma, and social determinants of health. Our research team created five interconnected domains to depict the key areas of healthcare that require improvement for people living with T1D: accessibility, appointments, shared decision‐making, support systems, and therapies.

We have also derived patient‐voiced actionable recommendations for clinicians, administrators, and policymakers for the improvement of T1D health services (Table 5). Used together, the framework and recommendations are intended to engage healthcare professionals and policymakers in meaningful dialogue to drive T1D clinical quality improvement.

We provide a comprehensive overview of our thematic analysis aligned with our QI framework, along with actionable recommendations. Table 4 summarizes the domains and corresponding themes identified across all interviews.

**Table 4:** Domains with themes and definitions.

Domains	Themes	Definitions
1. Accessibility	1.1 Centralized Hub	Centralized hub or “one‐stop‐shop” of information that patients, caregivers, and supporters of T1D can access.
	1.2 Mental Health	Difficulty in accessing mental health supports.
2. Appointments	2.1 Flexibility	After‐hours clinic access and alternative methods of appointments (telehealth, videoconference, etc.)
	2.2 Multidisciplinary	Provides a forum for discussion of divergent views on care strategies
3. Relationships	3.1 Enablers to Communication	Highly influences care perspectives, empowerment, and motivation
	3.2 Impacts of Medical Trauma	Lack of acknowledgement of lived experiences, miscommunication, and blame and shame.
	3.4 Power dynamics	Power dynamic between patient‐provider influence health‐seeking behaviours and the ability to play a role in one's care.
	3.5 Training & Education	Having a knowledgeable clinician provided a respectful and trusting environment critical to self‐management.
	3.6 Reproductive Health	Difficulty discussing pregnancy with experiences in authority and judgement.
4. Support Systems	4.1 DOC	Adjunct to clinical care through peer support
		Anonymous support provides an open dialogue
		Tacit knowledge sharing
		Potential source of mis‐ and disinformation, and negativity
	4.2 Family support	Childhood versus adulthood diagnosis
		Provide financial, emotional, and physical support
		Transition from paediatric to adult care
5. Therapies	5.1 Technology	Enabling access to the latest technology motivates better self‐management.
	5.2 Financial	Logistical barriers to appointments such as cost of travel, missed time from work, childcare, etc.
		Challenges in insurance coverage and affordability of T1D supplies and technology.
	5.3 Life Choices	T1D alters life choices in favour of paths that provide more security for T1D self‐management.

DOC, Diabetes Online Community, T1D, Type 1 Diabetes

#### 4.2.1 Domain 1: Accessibility

Theme 1.1 Centralized Hub for Patient Education

Accessibility centred on participants' access to T1D health services, which emphasized a lack of consistent information (P3) and the need for a centralized hub for information with credible and current resources for diabetes self‐management education and support (P23). These preferences were also highlighted by the need for experiential and interactive learning modalities.

I wish we had access to information in one location, like where you could get onto the internet, go straight to a website, and be able to access what you needed.

P3, F, 57, [7gpzg8sc]

If there was a central point where someone could go as newly diagnosed and from that hub there is all the information that they need.

P23, F, 58, [7gs32gp5]

Theme 1.2 Recognizing mental health burdens and lack of mental health supports

We identified mental health as a common factor associated with accessibility within participant discourse. Participants cited the value of mental health resources in the clinic (P37) and expressed frustration over the lack of coverage, which was also identified as a barrier to accessing mental health services. Being discharged from speciality care despite the persistent psychological burden of disease was also a driver of perceived challenges in mental health support (P14).

It can be very clinical, here's the numbers, here's the data, do this, do that, without really checking in on the mental health piece. [It] would be appreciated if there's just like a level of understanding that there's also like a human behind the data.

P37, F, 33, [7k9ty3ys]

I got my A1c down to the right level, I got, like dropped like a bad habit and no longer had mental health support, but that does not mean that the burden of diabetes goes away. That was frustrating because I didn't have the extended coverage for mental health support.

P14, F, 34, [7gpzgn8f]

#### 4.2.2 Domain 2: Appointments

Theme 2.1 Flexibility in clinical access

Participants valued the flexibility to attend appointments through virtual/telehealth and having concerns addressed through texts and calls between scheduled appointments (P10). Participants sought timely responses from clinicians (P33), but the challenges around appointments were identified through the difficulties in obtaining referrals, new providers, and covering travel costs to and from appointments (P19). Appointments that were redundant, particularly with routine follow‐ups, were regarded as wasteful and unnecessary (P41).

If I have issues, I call them outside of work hours. So, I'm very fortunate that I have very good access to my healthcare team when I need it.

P10, M, 37, [7gpzgj3x]

I saw the dietitian instead, who gave me all the answers I needed. If I was emailing her, she'd get back to me.

P33, M, 26, [7k9ty1h8]

There have been a few I've skipped just because I don't want to deal with the hassle and cost of trying to get there and back.

P19, F, 33, [7gq307dn]

I've wanted to blow off my endo appointments because I don't necessarily think that I am gaining anything and the only reason I do attend is because if I don't see [them] once a year, then I'll need a referral to see another.

P41, M, 63, [7k9ty7ms]

Theme 2.2 Enabling choice for multidisciplinary care

Participants valued multidisciplinary settings that facilitated holistic approaches to self‐management. Consistent messaging across clinicians was perceived as essential for continuity of care and shared decision‐making (P3). Appointments where the entire healthcare team was present together, allowed for open discussion of varying clinical opinions to constructively arrive at personalized strategies (P22).

We need a more well‐rounded experience so that when you come in, you can be seen by all on the same day, you can have all assessments done, the team can sit and discuss together.

P3, F, 57, [7gpzg80t]

They were on time, they had really thorough questions, and gave what felt to me like very solid feedback and reassuring advice.

P22, F, 22, [7gs32fbw]

#### 4.2.3 Domain 3: Relationships

Theme 3.1 Enablers and barriers to building trust in therapeutic patient‐provider relationships

Clinicians who were perceived as caring, compassionate, and non‐judgemental, fostered more collaborative care environments (P5). Those who prioritized the interconnectedness of biological, psychological, and social factors, provided a better experience overall (P31). Participants valued clinicians who were attentive, authentic, empathetic, supportive, and encouraging (P35).

He knew what he was talking about, and he cared. We ended up changing my treatment plan, I went off pump and started multiple daily injections, and that was something that I wanted to do for a long time. None of my previous doctors were open to discussing that.

P5, F, 28, [7gpzgb62]

I've had so many appointments where I have felt belittled, or that it's just been looking at numbers without understanding the full picture. Good appointments go beyond just the clinical numbers, they've involved talking about personal life and connecting in different ways.

P31, F, 37, [7j47prbd]

He was willing to listen, willing to learn, and willing to educate himself.

P35, F, 59, [7j47pxtl]

Clinicians who did not acknowledge the challenges of living with T1D, or the efforts being made to improve glycemic management would often leave the participant feeling judged and demoralized (P20). Overlooking patient efforts to cope with the burdens of self‐management hindered rapport building, whereas acknowledgement of patient efforts contributed to positive relationships (P3).

You feel ashamed around what your A1c is going to be, or you don't want to be bombarded with all the questions. It just feels like a lot when you go, it's a big judgement thing.

P20, F, 24, [7gq3085d]

We went into a lot of detail, and [they] said to me at the end of the appointment, ‘I really love having patients who take hold of their health'. [They] felt like I really wanted to make a difference in my own well‐being but also wanted to know all the clinical details.

P3, F, 57, [7gpzg8lr]

Participants appreciated when clinicians considered and discussed the life circumstances behind the lab results (P4). Clinicians who took the time to educate patients fostered greater trust and respect among participants (P36).

He really understood that diabetes was just one portion of my health, and that sometimes things have to adjust to make other things better.

P4, F, 31, [7gpzg9l9]

They're willing to go into the technical details and make me feel comfortable with that they're seeing whether it's based on my bloodwork and explain to me exactly is happening.

P36, M, 48, [7j47pz2z]

Being honest with the clinician was perceived as an important way to establish two‐way trust (P9).

There is that level of trust with a [clinician] where I just feel like the only way for the relationship to work is for me to be completely honest about myself

P9, M, 43, [7gpzggsf]

Theme 3.2 Recognizing the impact of medical trauma and burden of living with T1D

The time of diagnosis was recalled as particularly impactful, specifically regarding the negative aspects of T1D and the fear instilled in participants (P23). A misdiagnosis of T1D as T2D also contributed to a participant's negative perceptions of healthcare and whether people sought or did not seek future healthcare for T1D (P7). Medical trauma was rooted in experiences of ineffective communication, insufficient clinician knowledge about T1D, disregard for T1D lived experience, and paternalistic and authoritative approaches to care (P17). Traumatic healthcare encounters were also formed through experiences among hospital admissions for acute care, emergency services, or surgery that left individuals deprived of their autonomy to self‐manage their diabetes, resulting in distress (P35). Many of these experiences occurred during care encounters where participants, recalled being blamed, shamed, or having their T1D self‐management efforts disregarded (P15, P33).

Upon first diagnosis, you get all these doom and gloom fears that, Oh, my God, you have diabetes, you're going to lose a limb, you're going to go blind.

P23, F, 58, [7gs23g7h]

When I was diagnosed, I was not feeling well; it was quite sudden and I went to the doctor. He said go for blood tests tomorrow. I went and had a fasting glucose of 20 and they were like, ‘oh, okay, you're over 40 years old, its type 2 diabetes’. These appointments were spaced months apart and I went several times to try to get help because things were getting bad, I couldn't function. My spouse dragged me to the hospital to get a second opinion. Yeah… it was just… that was my first diagnosis which was a misdiagnosis due to age. Sorry, my hands are shaking; I haven't thought about this in a while.

P7, F, 49, [7gpzcyd]

The pump nurse bullied me into getting a pump because my numbers were great, and she kept insisting because I was under my mom's insurance. She contacted my mom and I was basically steamrolled. Anyway, I went on the pump, it was the worst 5 years of my diabetes, my numbers got worse.

P17, F, 39, [7gs321qm]

I have had moments where I go to the ER and they do bloodwork and come back and are like we think you have type 1 diabetes… as I lay there with a CGM on one arm and an insulin pump on the other.

P35, F, 59, [7j47pxxh]

Oh my god, I can still see the look on the woman's face, it was a female physician. Point blank, she stated to me word for word, why did you end up in the hospital? were you not taking your insulin? why do you continue to lie?

P15, F, 52, [7gpzgnq4]

I became ill one night and was throwing up every 30 min, I had no idea what was going on but I became very dehydrated. I went to the hospital in the small town I lived in and was admitted. The nurses started talking about me and how I was a bad diabetic and that I shouldn't be in there expecting them to fix my blood glucose. I deliberately had gone there for help, because I couldn't do it myself.

P33, M, 26, [7k9ty2by]

Theme 3.3 Recognizing power dynamics and lack of patient choice

Participants reported several issues with referral processes that created power struggles between patients and providers. Participants remained with their clinician due to the challenges in obtaining a new referral, even if they perceived that provider to be insufficient for their needs (P26). Clinicians who provided the opportunity for patients to be part of the medical team rather than medical subjects fostered a sense of autonomy that created trust and participant buy‐in (P8).

I don't think I have a choice but to trust the team that's in front of me, because it's hard enough to find care of any type at the moment, let alone specialized care.

P26, M, 45, [7gs32j1q]

I think they've done a good job of balancing, being my healthcare provider, but still giving me the autonomy or the options to pick from different things based on my understanding.

P8, M, 23, [7gpzgfwg]

Theme 3.4 Education for Healthcare Professionals

Participants felt that they were better able to trust clinicians who could provide up‐to‐date knowledge of T1D (P11). We found that participants cited clinician reluctance to discuss the use of unregulated technology for self‐management, such as Do‐It‐Yourself, aided insulin pump systems due to conflict with existing regulations or clinical guidelines (P34). Participants expressed frustration with a perceived lack of trained and knowledgeable clinicians for advanced T1D therapies (P15).

I think maybe that's what's missing from diabetes education is seeing each person as someone different and not trying to dismiss based on rules that are learned in med school.

P11, F, 29, [7gpzgkbd]

There are some doctors that I've spoken with who are really encouraging and would like some of their patients to be looping, and they can't outright say that because it's not approved.

P34, F, 32, [7j47pwqb]

More training is needed on how to understand my CGM data in relation to my pump data in relation to basal rates. Half the nurses have no clue what they're looking at and it is so frustrating.

P15, F, 52, [gpzgph2]

Theme 3.5 Having intentional and sensitive conversations on reproductive health

We found that positive clinician attributes enabled participants to feel more open to discussing sensitive topics. Specifically, participants indicated that diabetes‐related complications were challenging to discuss while others identified sex and reproductive health or mental health as particularly sensitive (P38). Women emphasized the importance of discussing reproductive intentions with their clinicians, noting that pregnancy heightened their motivation for self‐management (P34). However, we also observed that reproductive health was not addressed unless initiated by the patient in some instances (P33).

In my first meeting with him, my endocrinologist says to me ‘you cannot get pregnant without my permission'. I was like what does that even mean? I've had diabetes for 4 days.

P38, F, 55, [7k9ty50x]

With T1D, having a baby isn't all romance and sunshine. My team has always approached this with such care; we lost a pregnancy and that hurt. Those conversations are hard, there was a lot of tears. Going through that loss and checking in emotionally with me was huge.

P34, F, 32, [7j47pw48]

No one specifically had the conversation with me until I asked about it

P33, M, 26, [7k9ty2w6]

#### 4.2.4 Domain 4: Support Systems

Theme 4.1 Importance of fostering awareness of diabetes online communities

Participants expressed that diabetes online communities (DOCs) offered a platform for open discussion, often under the cloak of anonymity, which provided supplementary support as an adjunct to care (P20). DOCs were also helpful in validating experiences, providing troubleshooting tips, and daily self‐management recommendations (P41). Peer support benefitted those seeking a role model and helped others build resilience in learning different approaches to self‐management (P13). As reported by participants, the dissemination of misinformation and disinformation in DOCs was not discernibly higher than in mainstream social media. Participants were reluctant to try therapeutic strategies that were alternative or naturopathic without prior consultation with a qualified medical professional (P10). It was clear that participants were aware that insulin was essential for survival and were sceptical of alternative forms of therapy.

There are a lot of people advocating and sharing their experiences online, so it was a big eye‐opener for me.

P20, F, 24, [7gq308mg]

It's highly relatable because you can talk about a particular type of insulin that somebody might not be having as good an experience with and it's a great resource to have because you can always find somebody who's got an answer.

P41, M, 63, [7k9ty82b]

When I got diagnosed, I had some really bad role models, the way they took care of themselves. They were just very complacent. But when I started going online, you understand what works for them might not actually work for you.

P13, M, 24, [7gpzgmbc]

You have to take everything with a grain of salt, getting advice from others with diabetes is definitely different from getting advice from my endo.

P10, M, 37, [7gpzghzd]

Theme 4.2 Bolstering family support

Participants diagnosed in childhood recounted immense family support and management by parents and siblings in the form of emotional, physical, and financial support (P27). Participants diagnosed in adulthood cited less direct support from family members and less understanding of T1D and self‐management from loved ones (P37). Partners provided varying support, but the onus remained on participants to educate their partners and self‐manage given the unrecognized burdens of living with T1D (P12).

My mom was, you know, she was the gatekeeper, she definitely had to know everything.

P27, F, 53, [7gs32kbf]

Like my family is very supportive, but they will never truly understand what it's like, so it's just really nice when you find those people, and they just get it because they've lived it.

P37, F, 33, [7k9ty3wp]

My partner wakes up in the morning and wonders what time it is, I wake up in the morning and wonder what my blood sugar is at.

P12, F, 24, [7gpzglk6]

#### 4.2.5 Domain 5: Therapies

Theme 5.1 Enabling access to rapidly advancing technology

Participants reported technology as a central theme in T1D therapy, discussing various challenges and benefits of new insulins (P1), and the need for publicly funded access to diabetes technology (P13). Incorporating the latest technology was not always preferred, with some choosing to switch back to multiple daily insulin injections after trying an insulin pump therapy (P5). Importantly, CGMs were identified as essential for self‐management (P10). If faced with the choice to keep only one, a CGM was often preferred over an insulin pump due to improved glucose data, convenience over capillary blood testing, and the ability to proactively manage blood glucose (P4). The affordability of CGMs was identified as a major barrier to access (P2).

I'm big into technology, so if any new insulins or devices come out that is going to make managing easier, I want to be the first to get on those. Before my doctor would help me get on that, but now, I have to do the research myself.

P1, M, 33, [7gpzg76g]

I can count on the pump program, which is super important for me because it covers my supplies, which are very expensive, and I don't take advantage of that. It's something I'm diligent about and it's avoided me needing ER visits.

P13, M, 24, [7gpzg99j]

Costs are much lower on multiple daily injection than when I was on the pump. I was also unhappy having this thing attached to me. It was uncomfortable to keep the pump plus the needle.

P5, F, 28, [7gpzgbst]

The CGM, in my opinion, is actually the biggest piece to improving my health overall.

P10, M, 37, [7gpzghj3]

I think [the CGM] is by far the most useful tool you can have. Being able to see those things and how each thing in life affects me allows me to adjust [stay in range]. There is so much more peace of mind with the CGM than there is without one.

P4, F, 31, [7gpzg9pb]

I'm on the IPT program but one thing is that they don't cover CGM. To me, that would be great but I can't afford that it it's very, very expensive.

P2, M 76, [7gpzg7gf]

Theme 5.2 Managing T1D is expensive with many hidden costs, altering major life decisions

We observed financial burdens reported by participants, given the lack of public coverage for medications and CGMs and a recognized difficulty with self‐management due to those financial constraints (P34). Participants endorsed that investing tax dollars in publicly funded diabetes supplies and medication prevents downstream health system expenditures associated with emergencies and complications (P24).

There's this diabetes tax on everything… you earn less, eat less than someone beside you because you must pay so much for your life saving supplies all the time. I can't change to this job because I don't know what the benefits are going to be, or I can't go back to school because I might not have the coverage for my supplies that I have now, or I won't be able to afford a CGM.

P34, F, 32, [7j47ptzw]

If there is technology out there that would help you slow down or prevent complications, you're going to save healthcare a truckload of money.

P24, F, 67, [7gs32hc1]

The high costs associated with diabetes self‐management led to the rationing of supplies and risky self‐management behaviours (P9). Participants feared that their pump would be taken away from them or that they would be kicked out of the publicly funded programme if they did not manage their T1D well enough (P4). Participants with insurance coverage and access to technology praised the benefits of a publicly funded insulin pump therapy programme to improve their overall quality of life and ability to self‐manage T1D (P7). However, access to such programmes proved to be challenging for some due to the clinical criteria for pump eligibility (P33). One participant cited the frustration of having previously accessed and benefitted from T1D technology and then losing that access (P11).

There were a few times where I knew I had X number of strips left and when it was payday and I deliberately would either stretch my glucose strips out, and you know, check maybe twice a day, or even once a day, to make it to the end of the month.

P9, M, 43, [7g9pzgh8c]

A CGM though, if you took that away from me, I would be heartbroken. Because I can fix things before, they happen and understand trends to make decisions on my own because I can see the numbers in real time.

P4, F, 31, [7gpzg9p2]

[The Insulin Pump Program] has impacted me in the way that I feel I've been able to manage my T1D, almost to my best ability, because I haven't been overwhelmed by cost.

P7, F, 49, [7gpzgdzq]

I have been told that I must meet specific standards which make no logical sense. Before I could be signed off on this program, I had to get my A1c down, but you're limited to accessing a device that is meant to help control your blood sugar and get your A1c down.

P33, M, 26, [7k9ty1tp]

I know how well I can self‐manage on the Dexcom sensors; I had a better A1c, but I never had insurance to cover that and getting to see the difference between having and not having that access due to cost drastically changes my care, my mental health, my physical health, everything.

P11, F, 29, [7gpzgjln]

Lastly, participants discussed pursuing alternative career paths or higher education as a strategy for securing desirable employment and, therefore, better health insurance coverage. This was perceived as crucial for accessing technology and therapies that are deemed essential to living well with T1D (P4). Participants consistently said they would stay in unpreferred environments to ensure access to insurance coverage for T1D (P14).

I went to university to get a degree because I knew I needed a good job to pay for my diabetes. Most people look for a job that they love, I look for a job with good benefits.

P4, F, 31, [7gpzg99h]

You have to endure jobs that you didn't want to be in or environments that you didn't want to be in simply for the sole purpose of being able to afford your diabetes.

P14F, 34, [7gpzgmxm]

## 5 Discussion

Our findings show that individuals living with T1D value accessible, easy‐to‐navigate healthcare that provides consistent, up‐to‐date education, and personalized choices aligned with individual needs and values. Participants felt comfortable with clinicians who recognized the burdens of managing T1D and considered the socioeconomic and psychosocial context of self‐management to provide feasible treatment plans. These findings are consistent with the current recommended diabetes clinical practice guidelines [25, 26]. In contrast, poor or hostile communication that invoked blame or suggested a lack of clinician T1D knowledge, fostered medically traumatic experiences and often led to disengagement from healthcare.

### 5.1 Patient‐Centredness, Communication, and Empowerment

Participants frequently cited the importance of respect and agency in healthcare encounters, highlighting the need for clinicians to create welcoming spaces that enable patients to be active partners in their care. Person‐centred care is rooted in care relationships that incorporate individual needs, values, and preferences [27, 28, 29, 30]. The American Diabetes Association Standards of Care for T1D support collaborative clinical decisions that consider prognosis, individual preference, and financial well‐being [26]. Previous research shows that person‐centred care involves clinicians who dedicate time, support, and resources for T1D self‐management [31]. While person‐centred care is valued in clinical practice, its operationalization continues to be hindered by persistent stigma around diabetes and outdated medical education. More work is needed to foster trauma‐informed and empathy‐driven care, especially within primary and acute care settings.

Our findings also underscore the importance of patient‐centred care outside of office hours with participants reporting instances where timely access to medical guidance prevented episodes of diabetic ketoacidosis and emergency room visits. A large mixed‐methods investigation found that telehealth consultations prevented hospital admissions in 88% of diabetes patients at high risk for acute complications [32]. It is recommended that peer support networks continue to advocate with clinicians and health policymakers to expand alternative forms and access to health services [33]. Our findings highlight the often‐interchangeable relationship between the Appointments and Accessibility domains in our QI framework, suggesting that the way in which a person receives care, is closely linked to their access to it.

While multidisciplinary care approaches have been shown to improve metabolic markers, increase the use of diabetes technology, and enhance health‐seeking behaviours [34, 35], participants who managed T1D well under the guidance of a single provider did not perceive a need for additional multidisciplinary care. Adopting patient‐centred approaches, regardless of healthcare team composition, is important for high‐quality health services provision [36]. Communication breakdown leads to additional emotional distress, which discourages individuals from seeking care [37]. Team composition should reflect the patient's immediate and anticipated needs and evolve throughout the care relationship. This dynamic approach is crucial because the sense of helplessness, often experienced in traumatic situations, stems from a perceived lack of control over one's and well‐being [38].

Improved care for women who are navigating pregnancy is a documented gap in current T1D care [39, 40]. Targeted messaging has been shown to initiate conversations about pregnancy and T1D [41], while peer and family support can also help women alleviate the feelings of medicalization throughout their reproductive journey [42]. These approaches can be adapted to other elements of T1D clinical practice, such as conversations on sexual health, family planning, or vulnerable conversations about mental health and emotional well‐being.

#### 5.1.1 Building Resilience and Addressing the Impacts of Medical Trauma

Our findings reiterate the ongoing stigma individuals face in T1D health settings and highlight the importance of equipping individuals with the resources to better self‐manage T1D, contributing to disease acceptance, and trust in health systems [43, 44]. Specifically, educating clinicians to develop skills in emotional regulation and empathy are crucial for patient‐centred care [45]. We emphasize the urgency to consider and adopt the international consensus statement on challenging the harmful stigma and discrimination experienced by people living with T1D in clinical practice [46].

Our study provides evidence that T1D care often lacks trauma‐informed approaches, emphasizing the need for clinicians to discuss the intersecting factors between T1D and broader life challenges [5, 47]. Traumatic medical experiences eroded trust and discouraged future healthcare‐seeking behaviour by our participants. While this is documented in transitions from paediatric to adult T1D care settings [48, 49], it remains underexplored in adult T1D literature. Clinician education should include education in strengths‐based and trauma‐informed care [50], as new patient‐provider interactions provide opportunities to acknowledge and address the previous harm done within the medical system [38].

Misdiagnosis of T1D as T2D signals the difficulty clinicians face in differentiating diabetes subtypes, especially in the era of increasing global obesity [51]. Moreover, our findings suggest that misdiagnosis contributes to negative perceptions of T1D healthcare. Misdiagnoses of T1D has been previously shown to lead to higher rates of diabetic ketoacidosis [52] and reduced quality of healthcare related experiences [53]. Our findings suggest that there is more work needed to increase the diagnostic accuracy of T1D in adults.

#### 5.1.2 Clinical and Health Policy Impact

The next phase of this research will focus on implementing our framework as a reflective tool to complement existing QI initiatives, with an emphasis on fostering patient‐centred, trauma‐informed approaches to care. To ensure clinical impact, we will actively engage clinicians from our team in co‐developing realistic and feasible implementation strategies that maximize the uptake of our tools. While initiatives such as the T1D Exchange QI Collaborative [54] focus on standardizing care, our study prioritizes the responsive, empathetic, and contextually specific approaches needed to align with lived experiences of individuals with T1D. This study reinforces the challenges people with T1D face in affordable access to diabetes‐related technology which is critical to optimal T1D self‐management [55, 56]. Despite evidence that publicly funded programmes for T1D prove beneficial to taxpayers and people living with T1D, many regions still lag behind in adopting such policies [57]. Policymakers and clinicians must consider the rapidly evolving technology and evidence regarding the return on investment for these life‐saving therapies, as well as how health systems can more equitably provide access to this technology.

#### 5.1.3 Strengths and Limitations

The strengths of this research are rooted in our CBPR design in which co‐researchers living with T1D and clinicians providing T1D care were involved in the entire research process. The findings from our study have been distilled into a quality improvement framework (Figure 2) articulating the areas clinicians, health system administrators, and policymakers need to consider when improving care for individuals living with T1D along with prioritized actionable recommendations (Table 5). The framework can also be considered applicable to other chronic health conditions. We acknowledge that our study has some limitations, including the sample of an older, educated, more affluent, predominantly Caucasian, urbanized population with a longer duration of T1D. We recognize inadequate representation for those facing racialized or socioeconomic disparities. We were also not able to compare genders given insufficient data.

**Table 5:** Actionable Recommendations for Type 1 Diabetes Clinical Quality Improvement.

Recommendation	Healthcare providers	Administrators & policy makers
1. Create a Centralized Hub ‐ “One Stop Shop” for more equitable T1D navigation and access	Promote and contribute to the development of a centralized hub of information for T1D.	Develop a centralized hub of T1D information
		*For Patients:* how to navigate health insurance, latest therapies, peer support, research involvement.
		*For Clinicians:* Technology, insurance coverage, mental health resources, professional development, and research involvement.
		*For Supporters And Communities:* Diabetes stigma, general knowledge about T1D.
2. Increase Mental Health Resources in Practices	Build knowledge around mental health resources for T1D care teams and patients within your practice. This includes new avenues for training, advocating for clinic resources, and ongoing professional development.	Provide mental health support in T1D care by offering access to mental health professionals or training diabetes clinicians to guide patients in using mental health services.
3. Reduce out‐of‐pocket costs for T1D supplies and medication	Recognize that technology costs can be a barrier for some patients and create a supportive environment to adjust care based on their insurance and financial situation. Advocate for insurance coverage of CGMs and advanced therapies.	Subsidize access to continuous glucose monitoring technology as part of publicly funded healthcare.
4. Provide greater flexibility for clinical access	Offer personalized support by allowing patients to choose their T1D care team and decide who they see at each visit, recognizing that their needs may change over time. Address logistical barriers to appointments, whether in person, by phone, or by video.	Ensure adequate staffing to support patient load and needs. Offer telehealth or videoconference appointments based on patient needs and preference. Setup walk‐in appointments and/or after‐hours clinic access to prevent ER and hospital visits.
5. Build trusting relationships and acknowledge the resilience developed from living with T1D and social determinants of health	Start appointments by setting the patient's agenda. Be aware of your own biases and consider how the patient's experiences affect their self‐management. Focus on the patient's strengths and practice trauma‐informed care by acknowledging the challenges of T1D and any past medical trauma. Use supportive, non‐judgmental language, and respect the patient's knowledge of managing T1D. Check their health literacy and offer tools that are culturally and linguistically appropriate.	Promote person‐centred care by providing resources for ongoing education and training. Implement quality improvement initiatives to assess person‐centred care. Include training on effective and safe communication in T1D care in both student education programmes and continuing education for clinicians.

T1D, Type 1 Diabetes

## 6 Conclusion

The Reshape T1D study identifies areas for clinical quality improvement, voiced by patients through a CBPR approach. Our findings indicate that health services design should ensure that education and care for T1D is easy to access, free of stigma, consistent, and affordable. Further consideration is needed for how clinicians can appropriately implement the use of our QI framework and actionable recommendations to inform healthcare QI, but we encourage clinicians to begin considering this in their practice. The impact of medical trauma on health‐seeking behaviours is an important area that warrants further investigation.

## Author Contributions

**Jamie Boisvenue:** conceptualization, investigation, funding acquisition, writing–original draft, methodology, validation, visualization, writing–review and editing, software, formal analysis, project administration, data curation. **Youssef A Elezzabi:** methodology, writing–review and editing, formal analysis, conceptualization. **Kim Young:** conceptualization, methodology, validation, writing–review and editing, formal analysis. **Kathleen Gibson:** conceptualization, methodology, validation, writing–review and editing, formal analysis. **Heather Hinz:** conceptualization, methodology, validation, writing–review and editing, formal analysis. **Reid McClure:** conceptualization, methodology, validation, writing–review and editing, formal analysis. **Robyn Homulos:** conceptualization, methodology, validation, writing–review and editing. **Jude Spiers:** conceptualization, investigation, methodology, validation, writing–review and editing, formal analysis, project administration, supervision. **Peter Senior:** conceptualization, investigation, funding acquisition, methodology, validation, writing–review and editing, formal analysis, project administration, supervision, resources. **Roseanne Yeung:** conceptualization, investigation, funding acquisition, methodology, validation, visualization, writing–review and editing, formal analysis, project administration, supervision, resources.

## Ethics Statement

This research is approved by the University of Alberta Research Ethics Office (Pro000099177). Each participant provided informed consent and agreement of confidentiality before participating.

## Conflicts of Interest

JB received studentship funding from Diabetes Action Canada, the Government of Alberta, Alberta Diabetes Institute, Alberta Physician Learning Program, Alberta Strategy for Patient‐Oriented Research, and the University of Alberta School of Public Health for the duration of this project. YE received studentship funding from the Northern Alberta Clinical Trials & Research Centre for this project. KG, HH, RM, and YE received patient honoraria. KG, HH, KY, and JB received speaker fees related to the submitted work. PS is supported by the Alberta Academic Medicine Health Services Program and holds the Charles A Allard Chair in Diabetes Research outside of the submitted work. RY reports grants from the Alberta Health Services Diabetes Obesity and Nutrition Strategic Clinical Network and Alberta Diabetes Institute for this work and personal fees from Dexcom and Novo Nordisk outside the submitted work.

## Data Availability

Data that support the findings of this study are available on request from the corresponding author. The data are not publicly available due to privacy or ethical restrictions.
